# The Inhibition Effect of Epigallocatechin-3-Gallate on the Co-Aggregation of Amyloid-β and Human Islet Amyloid Polypeptide Revealed by Replica Exchange Molecular Dynamics Simulations

**DOI:** 10.3390/ijms25031636

**Published:** 2024-01-29

**Authors:** Xuhua Li, Yu Zhang, Zhiwei Yang, Shengli Zhang, Lei Zhang

**Affiliations:** 1 MOE Key Laboratory for Nonequilibrium Synthesis and Modulation of Condensed Matter, School of Physics, Xi’an Jiaotong University, Xi’an 710049, Chinayzws-123@xjtu.edu.cn (Z.Y.); zhangsl@xjtu.edu.cn (S.Z.); zhangleio@xjtu.edu.cn (L.Z.); 2 State Key Laboratory of Surface Physics, Department of Physics, Fudan University, 2005 Songhu Road, Shanghai 200438, China

**Keywords:** co-aggregation, epigallocatechin-3-gallate, replica exchange molecular dynamics simulation, amyloid-β, human islet amyloid polypeptide

## Abstract

Alzheimer’s disease and Type 2 diabetes are two epidemiologically linked diseases which are closely associated with the misfolding and aggregation of amyloid proteins amyloid-β (Aβ) and human islet amyloid polypeptide (hIAPP), respectively. The co-aggregation of the two amyloid proteins is regarded as the fundamental molecular mechanism underlying their pathological association. The green tea extract epigallocatechin-3-gallate (EGCG) has been extensively demonstrated to inhibit the amyloid aggregation of Aβ and hIAPP proteins. However, its potential role in amyloid co-aggregation has not been thoroughly investigated. In this study, we employed the enhanced-sampling replica exchange molecular dynamics simulation (REMD) method to investigate the effect of EGCG on the co-aggregation of Aβ and hIAPP. We found that EGCG molecules substantially diminish the β-sheet structures within the amyloid core regions of Aβ and hIAPP in their co-aggregates. Through hydrogen-bond, π–π and cation–π interactions targeting polar and aromatic residues of Aβ and hIAPP, EGCG effectively attenuates both inter-chain and intra-chain interactions within the co-aggregates. All these findings indicated that EGCG can effectively inhibit the co-aggregation of Aβ and hIAPP. Our study expands the potential applications of EGCG as an anti-amyloidosis agent and provides therapeutic options for the pathological association of amyloid misfolding disorders.

## 1. Introduction

Alzheimer’s disease (AD) and Type 2 diabetes (T2D) are the two most prevalent protein misfolding diseases (PMDs) [1]. The misfolding and aggregation of amyloid-β (Aβ) peptides are closely related with AD, and that of human islet amyloid polypeptide (hIAPP) peptides are tightly associated with T2D. Aβ is derived from the continuous cleavage of its amyloid precursor protein by β- and γ-secretase [2]. Distinct cleavage sites lead to the production of Aβ peptides ranging from 37 to 43 residues in length. Aβ peptides containing 40 and 42 residues are the two most prevalent isoforms [3]. It has been reported that Aβ42 exhibits greater cytotoxicity than Aβ40 [4]. The hIAPP peptide is a type of pancreatic β-cell hormone. It contains 37 residues with a disulfide bond between residues of Cys5 and Cys7 [5]. Epidemiological evidence indicates a non-independent correlation between the two PMDs [6,7,8,9,10,11,12,13,14,15,16,17]. Specifically, individuals with AD exhibit a heightened risk of developing T2D compared to the normal population, and vice versa [7,8,9,10,16,17]. Increasing evidence suggests that Aβ and hIAPP not only undergo self-aggregation into amyloid homo-oligomers and fibrils, but also have the capacity to co-assemble into hetero-aggregates, which may contribute to the molecular mechanism underlying the correlation between AD and T2D [18,19,20].

Exploring and developing effective inhibitors on amyloid aggregation is an important issue. In comparison to chemical drugs, which may entail side-effects and higher costs, compounds derived from natural products offer advantages such as increased stability, safety and enhanced biological compatibility. Many natural polyphenol compounds (e.g., epigallocatechin-3-gallate (EGCG) [21,22], resveratrol [23,24] and curcumin [25,26]) have been found to have effective inhibition against amyloid protein aggregation. Among these polyphenol molecules, EGCG, as an extract from green tea, has the strongest inhibition effect [27,28]. It has been widely reported that EGCG has the capacity to inhibit the amyloid aggregation of Aβ and hIAPP, and mitigate the toxicity associated with the two peptides by experiments [29,30,31,32,33,34,35,36]. Notably, a study on EGCG interacting with Aβ found that EGCG binds to Aβ monomers closely and then promotes them to grow into disordered and non-toxic aggregates rather than toxic oligomer or mature fibrils [33]. Additionally, EGCG exhibits the capability to disaggregate Aβ fibrils [34]. Research on EGCG interacting with hIAPP demonstrated that EGCG significantly inhibits the process of nucleation and fibrillation of hIAPP [35]. Molecular dynamics simulations revealed that EGCG inhibits the self-aggregation of both Aβ and hIAPP by reducing the inter-chain and intra-chain contacts of their dimers, inducing a conformational transformation from β-sheet to coil in their dimers [36,37]. 

Although EGCG has shown effective inhibition effect on amyloid aggregations of Aβ and hIAPP peptides, respectively, its potential to inhibit the co-aggregation of Aβ and hIAPP remains unclear. As amyloid proteins’ co-aggregation is implicated in the co-occurrence and associated development of two different PMDs, investigating the inhibition effect on the co-aggregation of amyloid proteins becomes equally crucial as that on their self-aggregation. Due to the complexity of amyloid protein aggregation, we cannot infer that EGCG can inhibit the co-aggregation of Aβ and hIAPP based solely on its ability to inhibit the self-aggregation of Aβ and hIAPP individually. Therefore, in this study, we utilized explicit solvent replica exchange molecular dynamics (REMD) simulation to examine the effect of EGCG on the co-aggregation of Aβ and hIAPP. Here, we chose dimer systems to investigate because oligomeric aggregates are the most toxic species, and dimers are the minimal toxic oligomer [38,39,40]. Our simulations reveal that EGCG significantly inhibits the co-aggregation of Aβ and hIAPP, reflected by the reduction in β-sheet formation within their amyloid core regions. Notably, polar residues and aromatic residues serve as the primary binding sites for EGCG on Aβ-hIAPP heterodimers. Through hydrogen-bond (H-bond), π–π and cation–π interactions, EGCG attenuates both inter-chain interaction between Aβ and hIAPP, as well as the intra-chain long-range interaction of Aβ and hIAPP within their heterodimers. Our study directly demonstrated the inhibition effect of EGCG on Aβ and hIAPP co-aggregation and elucidated the molecular mechanism underlying this effect, which contributes to a deeper understanding of the important role of EGCG molecules in combating co-aggregation-associated amyloidosis.

## 2. Results and Discussion

### 2.1. EGCG Significantly Diminishes the β-Sheet Propensity of Aβ-hIAPP Heterodimers

**Figure 1:**
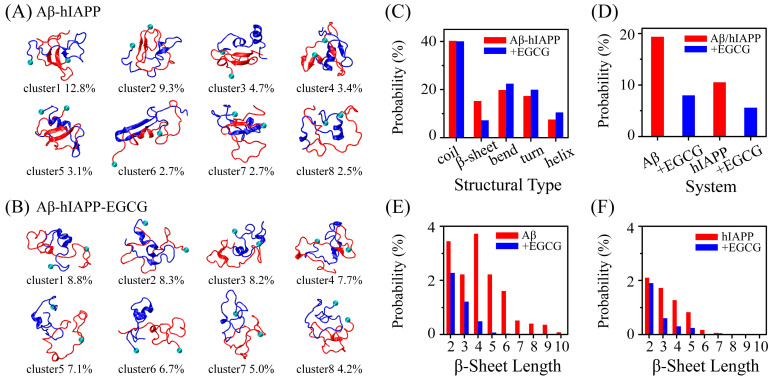
The effect of epigallocatechin-3-gallate (EGCG) on the β-sheet propensity of Aβ-hIAPP heterodimers. Representative conformations of the top-eight most-populated clusters of Aβ-hIAPP heterodimers (**A**) in the absence of EGCG and (**B**) in the presence of EGCG. Amyloid-β (Aβ) and human islet amyloid polypeptide (hIAPP) monomers are marked in red and blue, respectively. N-termini of Aβ and hIAPP are labeled with small balls. (**C**) The probability of each secondary structure of Aβ-hIAPP heterodimers in the absence and presence of EGCG. (**D**) The probability of β-sheet structures of Aβ and hIAPP in the absence and presence of EGCG. The probability of each β-sheet length of (**E**) Aβ and (**F**) hIAPP in the absence and presence of EGCG.

To assess the effect of EGCG on the structure of Aβ-hIAPP heterodimers, we conducted cluster analysis on Aβ-hIAPP heterodimers in the absence and presence of EGCG. Before analysis, we checked the convergence of our REMD simulation. All subsequent analyses are based on the converged data, and detailed information can be found in the Convergence analysis and Figure S1 in Supplementary Materials. The representative conformations of the top-eight most-populated clusters of Aβ-hIAPP heterodimers reveal that in presence of EGCG, the conformations of Aβ-hIAPP heterodimers are more disordered with reduced β-sheet structures (Figure 1A,B). Long β-hairpin structures (cluster1 and cluster6) and β-sheets formed between Aβ and hIAPP (cluster4 and cluster7) are shown in the Aβ-hIAPP system, whereas they are almost absent in the Aβ-hIAPP and EGCG complex system (Aβ-hIAPP-EGCG) (Figure 1A,B). To quantitatively characterize the structural differences, we calculated the probability of each typical secondary structure of Aβ-hIAPP heterodimers both in the absence and presence of EGCG, including coil, β-sheet, bend, turn and helix. As depicted in Figure 1C, the probability of coil is comparable in both the pure Aβ-hIAPP system and the Aβ-hIAPP-EGCG system. Notably, the β-sheet probability of heterodimers in the Aβ-hIAPP-EGCG system is obviously lower, and the probabilities of bend, turn and helix structures are higher than those in Aβ-hIAPP system without EGCG. Furthermore, we calculated the β-sheet probability separately for Aβ and hIAPP in both systems. The results indicate that the probabilities of β-sheet structures for both peptides are lower in the presence of EGCG than those without EGCG (Figure 1D). Additionally, regarding the distribution of β-sheet length, both Aβ and hIAPP exhibit shorter β-sheet length in the presence of EGCG (Figure 1E,F). All the results demonstrate that EGCG significantly diminishes the β-sheet propensity of Aβ-hIAPP heterodimers. Given that the β-sheet formation is crucial for the fibrillization of amyloid proteins, the observed reduced β-sheet probability strongly suggests that EGCG effectively inhibits the co-aggregation of Aβ and hIAPP.

### 2.2. EGCG Attenuates the Aggregation Propensity of Both Aβ and hIAPP in Their Amyloid Core Regions

**Figure 2:**
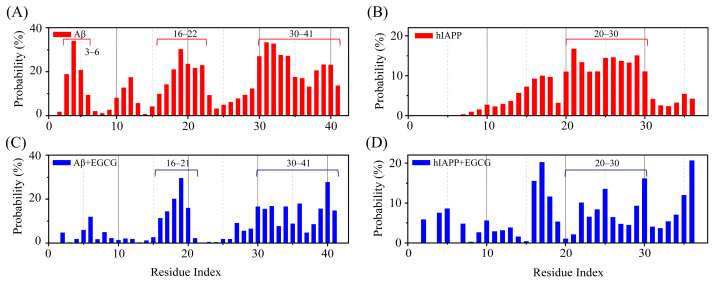
The inhibition effect of EGCG on the β-sheet formation in amyloid core regions of Aβ and hIAPP. The probability of β-sheet on each residue of (**A**) Aβ and (**B**) hIAPP in the absence of EGCG, and (**C**) Aβ and (**D**) hIAPP in the presence of EGCG.

In the Aβ-hIAPP heterodimer system, the regions with high β-sheet probability of Aβ are predominantly located in its amyloid core regions, including N-terminal region E3–H6, the central hydrophobic core region (CHC) K16–E22 and the C-terminal hydrophobic region I30–I41 (Figure 2A). Similarly, the region with high β-sheet probability in hIAPP is primarily situated within its amyloid core region N20–T30 (Figure 2B). In the Aβ-hIAPP-EGCG system, the β-sheet probabilities of the residues within these amyloid core regions are markedly decreased (Figure 2C,D). For Aβ peptide, the regions with high β-sheet propensity remain similar to those in the absence of EGCG. Although the β-sheet probabilities in N-terminal region E3–H6 almost disappear, those in the CHC region and C-terminal region are weakened greatly but not completely eliminated (Figure 2C). For hIAPP, the β-sheet distribution is notably altered, with the β-sheet probability being weakened in the amyloid core region N20–T30 (Figure 2D). The observed weakening effect of EGCG on the β-sheet formation in amyloid core regions of both Aβ and hIAPP further supports the inhibition effect of EGCG on the co-aggregation of Aβ and hIAPP.

### 2.3. Polar and Aromatic Residues Serve as the Primary Binding Sites for EGCG within Both Aβ and hIAPP

**Figure 3:**
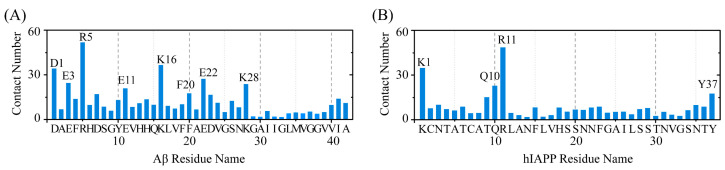
The binding sites for EGCG with Aβ and hIAPP peptides. The contact number between EGCG and each residue of (**A**) Aβ and (**B**) hIAPP.

To elucidate the molecular mechanisms underlying the inhibition effect of EGCG on the co-aggregation of Aβ and hIAPP, we first investigated the binding site between EGCG and Aβ-hIAPP heterodimers. The residue-based contact number between EGCG and Aβ/hIAPP peptide in heterodimers is calculated (Figure 3). In the case of the Aβ peptide, residues with a high contact number with EGCG are primarily located in the negatively charged residues D1, E3, E11 and E22, and the positively charged residues R5 and K28, as well as aromatic residue F20 (Figure 3A). A similar binding pattern is also observed for hIAPP peptides, where high-contact-number residues include positively charged residues K1, R11 and aromatic residue Y37 and polar residue of Q10 (Figure 3B). All these residues with high contact numbers are polar and aromatic residues, which also play a crucial role on the formation and stabilization of their homogeneous amyloid fibrils [41,42,43]. These polar and aromatic residues serve as the primary binding sites for EGCG with Aβ and hIAPP, suggesting that the binding sites of the two proteins with EGCG have a competitive relationship with protein-protein binding sites in their homogeneous fibrils and thus hinders the fibrilization of Aβ and hIAPP.

### 2.4. The Interactions between EGCG and Aβ-hIAPP Heterodimers Primarily Involve H-Bond Interaction, π–π Stacking and Cation–π Interaction

**Figure 4:**
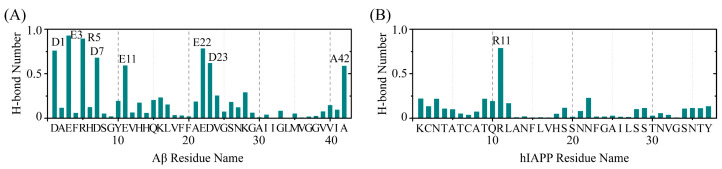
The H-bond interaction analysis of EGCG and Aβ-hIAPP heterodimers. The H-bond number between EGCG and each residue of (**A**) Aβ and (**B**) hIAPP.

Considering the primary binding sites for EGCG are polar residues and aromatic residues, capable of interacting with EGCG through H-bond interaction, π–π stacking and cation–π interaction, we then evaluated these interactions between EGCG and Aβ-hIAPP heterodimers based on residues. The H-bond number between EGCG and each residue of Aβ and hIAPP is shown in Figure 4. As we expected, residues exhibiting a high H-bond number binding with EGCG are located in polar residues of these binding site residues for both Aβ and hIAPP, primarily including D1, E3, R5, D7, E11, E22 and D23 of Aβ and R11 of hIAPP.

**Figure 5:**
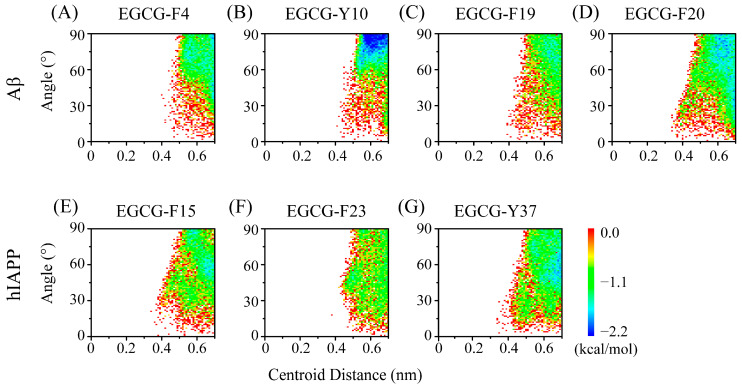
The π–π stacking interaction analysis of EGCG and Aβ-hIAPP heterodimers. PMFs (in kcal/mol) as a function of the centroid distance and the angle of two aromatic: EGCG rings vs. aromatic residues of (**A**–**D**) Aβ and (**E**–**G**) hIAPP.

We then quantified the π–π stacking interaction between EGCG and aromatic residues of Aβ and hIAPP peptides. As can been seen in Figure 5, EGCG exhibits π–π stacking interactions with all aromatic residues of Aβ and hIAPP. In the case of Aβ peptide, although EGCG and Y10 have a minimum-energy basin, the π–π stacking between EGCG and F20 is strongest among the four residues (Figure 5A–D), which is consistent with the higher contact number between EGCG and F20 (Figure 3A). Regarding hIAPP, F15 and Y37 present stronger π–π stacking interactions among the three residues, with Y37 displaying a more pronounced parallel π–π stacking configuration than F15 (Figure 5E–G).

**Figure 6:**
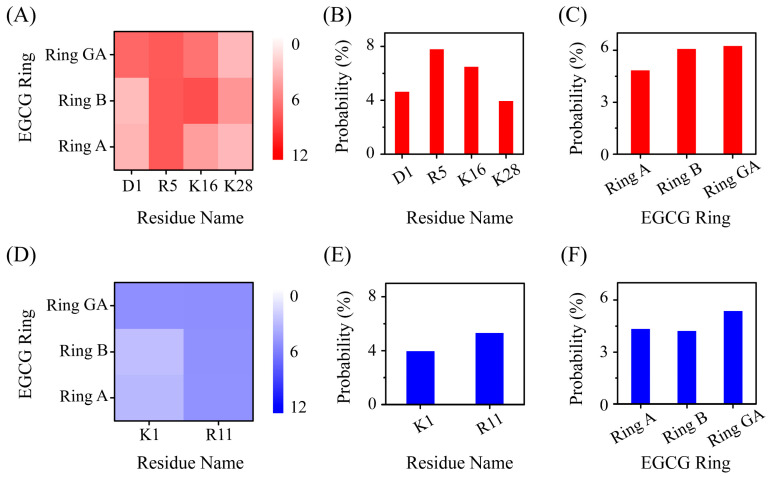
The cation–π interaction analysis of EGCG and Aβ-hIAPP heterodimers. Cation–π probability maps between three aromatic rings of EGCG and positively charged residues of (**A**) Aβ and (**D**) hIAPP. The cation–π interaction probability for (**B**) positively charged residues of Aβ with EGCG and (**C**) for three rings of EGCG with Aβ. The cation–π interaction probability for (**E**) positively charged residues of hIAPP with EGCG and (**F**) for three rings of EGCG with hIAPP.

We also calculated the cation–π interaction between all positively charged residues of Aβ/hIAPP peptide and three aromatic rings of EGCG. An EGCG molecule comprises three aromatic rings labeled ring A, ring B and ring GA (Figure S2) [44]. The cation–π interaction between positively charged residues and each aromatic ring in EGCG are mapped in Figure 6. In terms of positively charged residues, R5 and K16 of Aβ peptide display a high probability of interaction with EGCG through cation–π interaction, and R11 of hIAPP presents a high probability with EGCG (Figure 6B,E). Regarding three aromatic rings of EGCG, we found that ring B and ring GA display a higher probability of forming cation–π interactions with positively charged residues (Figure 6C,F), indicating that ring B and ring GA play a more important role in interacting with Aβ-hIAPP heterodimers. It is reported that an aromatic ring carrying three or more oxhydryls is a crucial structure motif which can effectively interact and then inhibit amyloid protein fibrillation [45,46]. Among the three rings in an EGCG molecule, ring B and ring GA have three oxhydryls in their aromatic rings, while ring A has two. To compare the importance of the three aromatic rings within EGCG, we calculated the contact number and H-bond number between the three aromatic rings of EGCG and Aβ-hIAPP heterodimers. In terms of both the contact number and the H-bond number, ring B and ring GA have higher quantities than ring A (Figure S3), which further emphasizes the effectivity of ring B and ring GA within EGCG for inhibiting amyloid aggregation.

### 2.5. EGCG Attenuates Both Inter-Chain Interaction between Aβ and hIAPP, as Well as the Intra-Chain Long-Range Interaction of Their Respective Peptides within Aβ-hIAPP Heterodimers

**Figure 7:**
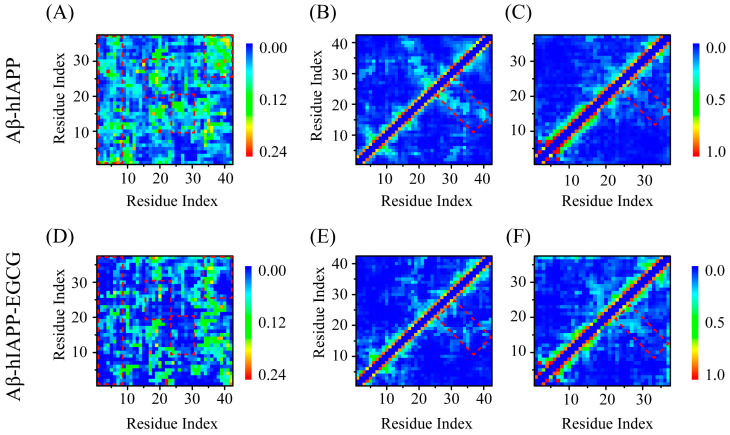
The inter-chain and intra-chain interaction analysis of Aβ-hIAPP heterodimers. The inter-chain contact maps between Aβ and hIAPP (**A**) in the absence of EGCG and (**D**) in the presence of EGCG. The intra-chain contact maps of (**B**) Aβ and (**C**) hIAPP in the absence of EGCG. The intra-chain contact maps of (**E**) Aβ and (**F**) hIAPP in the presence of EGCG.

To reveal how EGCG inhibits Aβ and hIAPP co-aggregation via the above-mentioned H-bond, π–π stacking and cation–π interactions, we examined the contact networks between Aβ and hIAPP, as well as within Aβ and hIAPP peptides. Regarding the inter-chain interaction between Aβ and hIAPP, we observed that the contact probability is reduced in several regions in the presence of EGCG, including Aβ_1–7_ and hIAPP_1–37_, Aβ_16–22_ and hIAPP_18–30_, Aβ_24–32_ and hIAPP_8–18_, and Aβ_33–42_ and hIAPP_25–37_ (represented by dotted areas in Figure 7A,B). The residues within these regions encompass the EGCG binding site residues, implying that EGCG directly attenuates the inter-chain interaction between Aβ and hIAPP by binding with them. The observed weakening of the inter-chain interaction provides direct evidence that EGCG inhibits the co-aggregation of Aβ and hIAPP. We also calculated the intra-chain interaction within Aβ and hIAPP peptides. In the case of Aβ, EGCG significantly weakens the long-range interaction within the peptide chain, particularly between residues Aβ_15–25_ and Aβ_25–42_ (Figure 7B,E). For hIAPP, although the local interactions are enhanced in the presence of EGCG, the long-range interaction is weakened to some extent (Figure 7C,F). These diminished intra-chain interactions would, on one hand, inhibit the co-aggregation between Aβ and hIAPP and, on the other hand, hinder their own self-aggregation.

## 3. Materials and Methods

### 3.1. System Modeling

The disordered coil-rich monomers of Aβ and hIAPP peptides were utilized to construct the Aβ-hIAPP heterodimer system (Aβ-hIAPP), as well as the Aβ-hIAPP and EGCG complex system (Aβ-hIAPP-EGCG). The hIAPP peptide contains a disulfide bridge between Cys2 and Cys7 and has an amidated C-terminus. The initial structures of Aβ-hIAPP heterodimers are obtained from our previous study [47]. Prior ThT fluorescence assays and toxicity experiments have demonstrated that a five-fold molar excess of EGCG is effective in completely suppressing the amyloid fibril formation of Aβ or IAPP and has been proven sufficient to inhibit the resulting cytotoxicity [34,48,49]. Therefore, we chose 5:1 as the molar ratio of EGCG:Aβ-hIAPP for our study. The initial conformations of the Aβ-hIAPP-EGCG system were created by introducing ten EGCG molecules into the Aβ-hIAPP heterodimer system (Figure S4). Herein, we utilized a Ramachandran plot to evaluate the structure validity of Aβ-hIAPP heterodimers. In the Ramachandran plot, the blue color represents the most favorable regions for α-helix and β-sheet, while the green color represents the less favorable regions, and the white color denotes disallowed regions where residues exhibit a loss of secondary structure [50]. Figure S5 displays Ramachandran plots depicting initial structures of Aβ-hIAPP heterodimers within the Aβ-hIAPP-EGCG system. Most residues are located within energetically allowed regions, indicating that the configurations of these heterodimers, used as initial structures in our simulation, are reasonable. The atomic structure of EGCG was taken from the PubChem library (CID: 65064). Figure S2 illustrates the chemical structure of an EGCG molecule. Referring to our previous MD simulation studies [51,52], the partial charges for each atom of EGCG were determined by fitting to quantum mechanical calculated potentials, which are obtained by using the Ambertools package [53]. To enhance the sampling, a total of sixteen different initial structures of Aβ-hIAPP-EGCG systems were constructed by changing the relative positions of the two peptide chains (Figure S4). Both systems were placed into periodic cubic simulation boxes, which were large enough for peptide translation and rotation freely without interacting with their periodic images. The size of the cubic box was set to 7.5 × 7.5 × 7.5 nm^3^ for both the Aβ-hIAPP and Aβ-hIAPP-EGCG systems, each filled with 130,94 and 12,899 water molecules, respectively. The Aβ-hIAPP system comprised 40,522 atoms, and the Aβ-hIAPP-EGCG system contained 40,447 atoms. Na^+^ and Cl^−^ were added to neutralize the systems and to mimic the physiological salt condition, with a NaCl salt concentration of 150 mM for each system. The modeling details of the Aβ-hIAPP and Aβ-hIAPP-EGCG systems are shown in Table 1.

### 3.2. Simulation Details

A 500 ns REMD simulation was conducted for the Aβ-hIAPP heterodimer system and a 600 ns REMD simulation for the Aβ-hIAPP-EGCG complex. For each system, a total of 48 replicas were employed, with temperatures exponentially dispersed between 308 K and 400 K. As a result, the combined simulation time for each system was 14.4 µs. The temperature list for the Aβ-hIAPP heterodimer system is referenced from our previous study [47], and that of the Aβ-hIAPP-EGCG system is shown in Table S1. We chose the trajectory of the replica at 310.00 K for data analysis, in line with the physiological temperature. The attempted swap time between two neighboring replicas was set at 2 ps. The average acceptance ratio is ~20% for each system, which has demonstrated to be efficient in numerous REMD simulation studies [54,55,56]. Both REMD simulations were performed by using the GROMACS 2018.8 software package [57,58]. The atomic interactions of proteins were calculated using the Amber99SB-ILDN force field [59], and the transferable intermolecular potential with three points (TIP3P) water model [60] was used to describe the solvent. The Amber99SB-ILDN force field is a well-established choice for investigating amyloid protein aggregation [56,61,62]. Moreover, several studies conducted comparisons among common biomolecular force fields, including Amber99SB-ILDN, Gromos53a6, OPLS-AA/L, CHARMM22, etc. These investigations consistently conclude that the simulation results conducted under the Amber99SB-ILDN force field exhibit stronger agreement with experimental findings than other force fields, particularly when simulating intrinsically disordered proteins [63,64,65,66]. All simulations were conducted under the isothermal–isobaric ensemble. The temperature of each simulation was maintained at a constant value using the velocity rescaling coupling method [67]. The protein and nonprotein groups were separately coupled to an external heat bath with a 0.1 ps relaxation time. The pressure was kept at 1 bar with a coupling constant of 2 ps using the Parrinello–Rahman method [68]. Constraints between hydrogen atoms and other heavy atoms were applied for water molecules using the SETTLE algorithm [69] and for proteins using LINCS algorithm [70], which allows a 2 fs integration time step using the Verlet integrator. Electrostatic interactions were calculated using the particle-mesh Ewald method [71] with a real-space cut-off of 1.0 nm. The same cut-off was used for van der Waals interactions. Periodic boundary conditions were applied in all simulations. These methods and parameters have been widely used in previous simulation studies [47,55,62,72,73,74]. The simulation details for REMD simulations of the two systems were listed in Table 1.

### 3.3. Analysis Methods

We analyzed the simulation trajectories using multiple parameters, which encompassed the radius of gyration, H-bond number, secondary-structure content and β-sheet length. Additionally, contact number, contact probability maps, π–π stacking and cation–π probability were also calculated. These analytical processes were executed using both our in-house algorithms and the tools implemented in GROMACS. Here, the secondary structure was identified utilizing the Define Secondary Structure of Protein (DSSP) program [75]. The average percentage of each type of secondary structure was determined, along with the residue-based probability distribution of these secondary structures. Terminal residues at the N- and C-termini of each chain were ignored in residue-based β-sheet/helix/bend/turn probability calculation, as they are always in random coil conformation. For cluster analysis, the Daura method [76] was employed with a C_α_-RMSD cut-off of 0.35 nm. An atomic contact was defined when the aliphatic atoms of two nonsequential residues were within 0.54 nm or when any other nonhydrogen heavy atoms of two nonsequential residues were within 0.46 nm [52,55]. π–π stacking interactions between two aromatic rings were identified when their centroid distance fell within 0.7 nm [42]. A cation–π interaction was established when the minimum distance between the N atom in the NH^3+^ group and the aromatic ring plane was less than 0.7 nm. The conformations of Aβ-hIAPP co-aggregates in the absence/presence of EGCG were visualized using the Visual Molecular Dynamics (VMD) program [77].

## 4. Conclusions

We investigated the inhibition effect of EGCG on Aβ and hIAPP co-aggregation by using the all-atom enhanced-sampling REMD simulations. The findings indicate a significant reduction in the β-sheet contents of Aβ-hIAPP heterodimers induced by EGCG. Notably, the diminished β-sheet regions are predominantly located within amyloid core regions of Aβ and hIAPP, suggesting a direct inhibition of their subsequent amyloid fibrillation. The EGCG binding sites are predominantly in the polar and aromatic residues of Aβ and hIAPP peptides with strong H-bond interaction, π–π stacking and cation–π interaction. Through these interactions, EGCG effectively attenuates the inter-chain interaction between Aβ and hIAPP, as well as the intra-chain long-range interaction of Aβ and hIAPP peptides within their heterodimers. One limitation of our study is the absence of a comparative analysis of the inhibition effects between EGCG and other tea polyphenol extracts, such as EGC, ECG or theaflavin, which could be further explored in subsequent studies. Our study reports the inhibition effect of EGCG on Aβ-hIAPP co-aggregation and elucidates the molecular mechanisms behind this effect, which expands the potential application of EGCG from targeting single amyloid aggregations to addressing co-aggregation involving different amyloid proteins. Additionally, the elucidation of the molecular mechanisms underlying the inhibition effect serves as a theoretical foundation for the development of treatment strategies targeting the pathological association of amyloid misfolding disorders.

## Figures and Tables

**Table 1:** Modeling and simulation details for REMD of Aβ-hIAPP and Aβ-hIAPP-EGCG systems.

System	Aβ-hIAPP	Aβ-hIAPP-EGCG
Number of replicas	48	
Temperature range	308.20–400.00 K	
Simulation time	500 ns	600 ns
Number of atoms	40,522	40,447
Number of water molecules	13,094	12,899
Box size	7.5 × 7.5 × 7.5 nm^3^	
Number of EGCG	---	10

## Data Availability

The data in this study are readily available upon reasonable request to the corresponding author.

## Supplementary Materials

The supporting information can be downloaded at: https://www.mdpi.com/article/10.3390/ijms25031636/s1.
