# Supplementary material for: The Inhibition Effect of Epigallocatechin-3-Gallate on the Co-Aggregation of Amyloid-β and Human Islet Amyloid Polypeptide Revealed by Replica Exchange Molecular Dynamics Simulations
Source: Int J Mol Sci. 2024 Jan 29;25(3):1636. doi: 10.3390/ijms25031636 (PMC10855639; doi:10.3390/ijms25031636)
Supplement: Supplementary file 1 [file ijms-25-01636-s001.zip › ijms-2834263-supplementary.pdf]

Supporting Information

The Inhibition Effect of Epigallocatechin-3-Gallate on the Co-Aggregation of Amyloid-β and Human Islet Amyloid Polypeptide Revealed by Replica Exchange Molecular Dynamics Simulations

Xuhua Li[a,b]*†, Yu Zhang[a]†, Zhiwei Yang[a], Shengli Zhang[a], Lei Zhang[a]

a. MOE Key Laboratory for Nonequilibrium Synthesis and Modulation of Condensed Matter, School of Physics, Xi'an Jiaotong University, Xi'an 710049, China
b. State Key Laboratory of Surface Physics, Department of Physics, Fudan University, 2005 Songhu Road, Shanghai, 200438, China.

The authors declare no competing financial interest.

*Corresponding author: Email: xuhuali@xjtu.edu.cn
†These authors contributed equally to this work.

Convergence analysis

Prior to data analysis, we first assessed the convergence of the REMD simulation for Aβ-hIAPP and EGCG complex (Aβ-hIAPP-EGCG) system by several aspects, including the replica exchange efficiency, the probability density function (PDF) of structural parameters over two time-intervals (400-500 ns and 500-600 ns) and the probability of typical secondary structures based on each residue within same time-intervals. As can be seen in Figure S1A and B, all replicas have traversed at 310 K during the 500-ns REMD simulations and the replica initially at 310 K sufficiently visited the entire temperature space as well. The PDF of the radius of gyration (Rg) and H-bond number within two time-intervals exhibit significant overlaps (Fig. S1C, D). Moreover, the probability of each secondary structure and the probability distributions of three typical secondary structures (coil, β-sheet and helix) for each residue exhibit a notably consistent agreement within the two time-intervals. All these findings collectively indicated that the REMD simulation achieved good convergence during the 400-600 ns period, and consequently, all analyses for the Aβ-hIAPP-EGCG system are based on the last 200 ns.

One supplementary table

Table S1. Temperature (K) list used in the 48-replica REMD simulations of Aβ-hIAPP-EGCG complex. The temperatures for each replica are located below the corresponding replica index, and are marked in a grey background.

1	2	3	4	5	6	7	8
308.20	310.00	311.81	313.62	315.44	317.27	319.11	320.94
9	10	11	12	13	14	15	16
322.79	324.65	326.51	328.38	330.27	332.16	334.05	335.96
17	18	19	20	21	22	23	24
337.82	339.79	341.71	343.65	345.59	347.54	349.50	351.47
25	26	27	28	29	30	31	32
353.45	355.43	357.42	358.92	360.93	362.94	364.96	366.99
33	34	35	36	37	38	39	40
369.03	371.08	373.14	375.20	377.28	379.36	381.44	383.54
41	42	43	44	45	46	47	48
385.65	387.77	389.83	391.96	394.11	396.26	498.42	400.00

Five supplementary figures

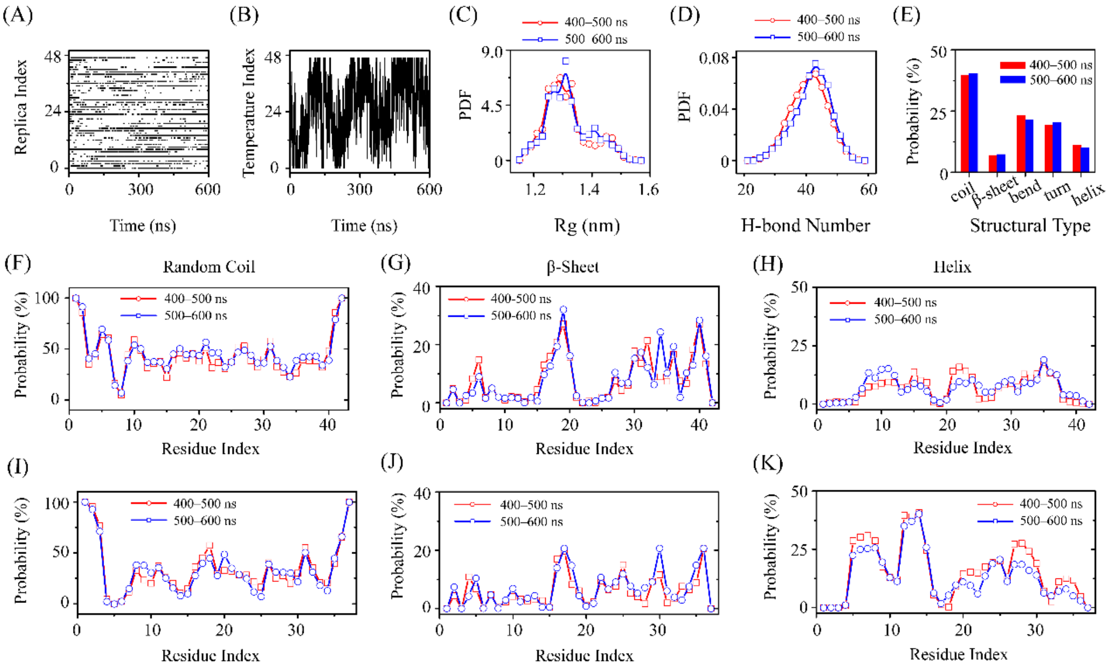

Figure S1. Simulation convergence assessments for the Aβ40-Aβ42-EGCG system. The time evolution of (A) the replica index simulating under temperature of 310 K and (B) the replica initially at 310 K swapping in temperature space; the probability density function (PDF) of (C) Rg and (D) total H-bond number; (E) the average probability of each dominant secondary structure (including random coil, β-sheet, bend, turn and helix); (F-H) the random coil, β-sheet and helix propensity of each residue of Aβ42; (I-K) the random coil, β-sheet and helix propensity of each residue of hIAPP. The convergence was checked using the data generated within the time intervals of 400-500 ns and 500-600 ns. The analytic processes were executed using the tools implemented in GROMACS software and DSSP program.

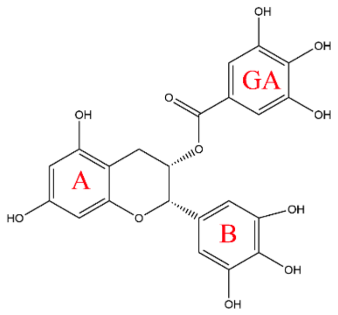

Figure S2. The chemical structure of EGCG.

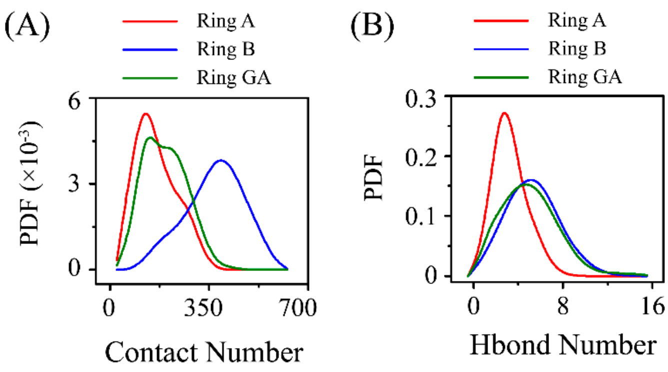

Figure S3. The interaction between Aβ-hIAPP heterodimers and EGCG analysis. Probability distribution function (PDF) of (A) contact number and (B) H-bond number for Aβ-hIAPP heterodimers and three distinct aromatic rings of EGCG. The analysis of the number of contact and H-bond was executed using in-house algorithms and the tools implemented in GROMACS software, respectively.

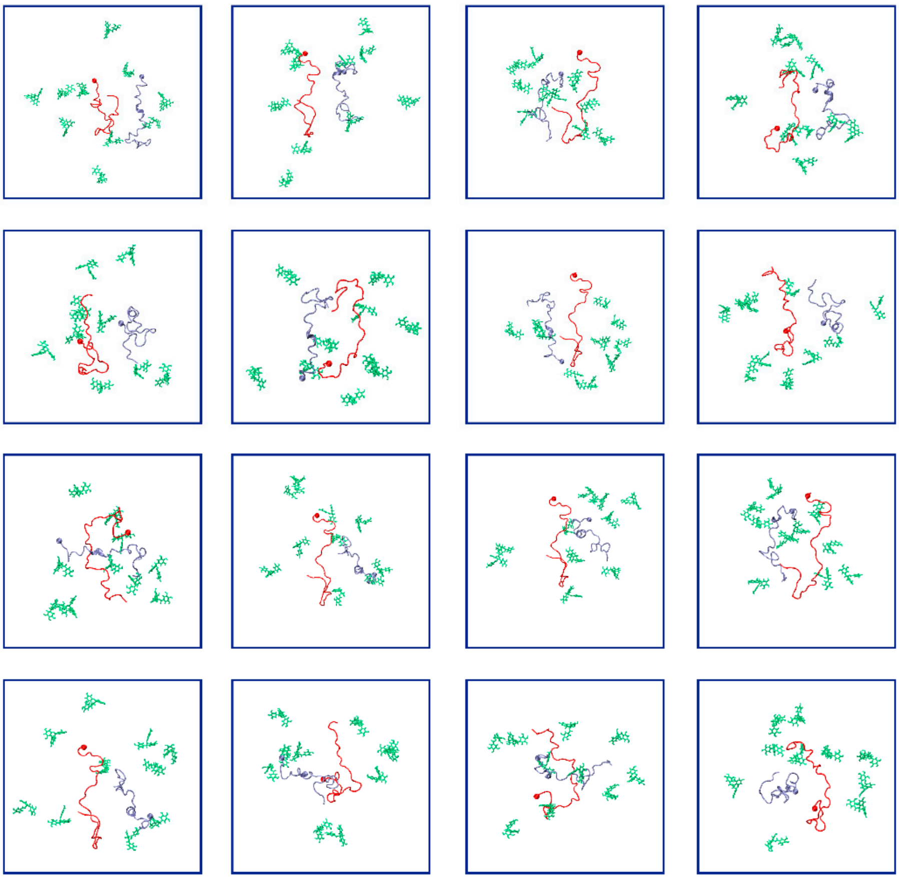

Figure S4. The initial structures of Aβ-hIAPP-EGCG complex. The N-terminus of peptides are labeled with small balls. Aβ, hIAPP and EGCG molecules are colored in red, blue and green, respectively. The peptides are shown in NewCartoon representation and EGCG molecules are shown in Licorice representation using VMD program. To

clearly represent Aβ, hIAPP and EGCG molecules, water molecules are hidden in these diagrams.

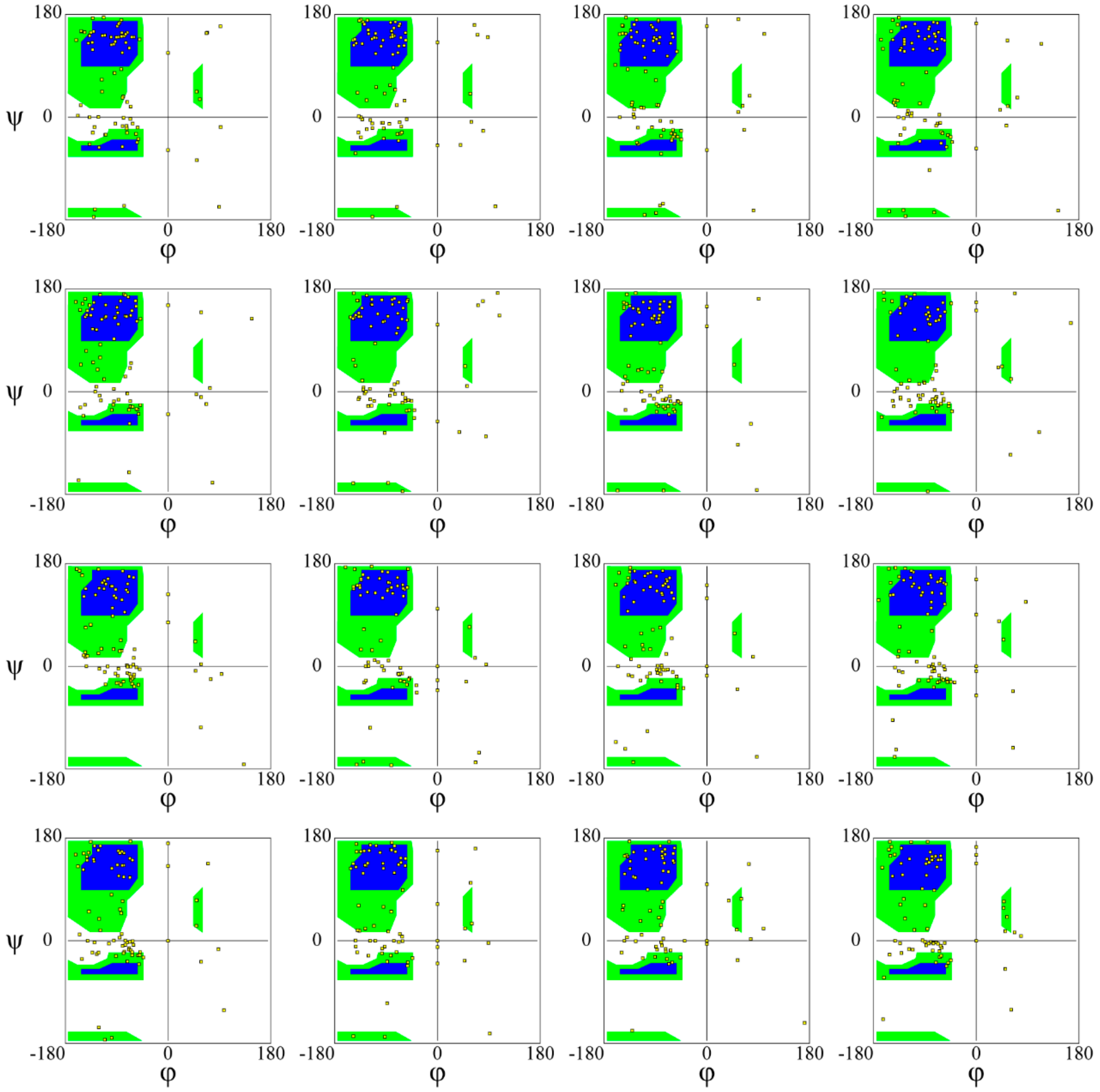

Figure S5. Ramachandran plots of initial structures of Aβ-hIAPP heterodimers within Aβ-hIAPP-EGCG complexes. These images were generated using VMD program.
